# Recovery of carbon and cryolite from spent carbon anode slag of electrolytic aluminum by flotation based on the evaluation of selectivity index

**DOI:** 10.3389/fchem.2022.1025990

**Published:** 2022-10-10

**Authors:** Yemin Wang, Xuexia Wang, Muhammad Bilal

**Affiliations:** ^1^ Department of Mining Engineering, Shanxi Institute of Technology, Yangquan, China; ^2^ Department of Mining Engineering, Balochistan University of Information Technology, Engineering and Management Sciences (BUITEMS), Quetta, Pakistan

**Keywords:** carbon anode slag, grinding flotation, carbon, cryolite, selectivity index

## Abstract

One of the main electrolytic aluminum production costs is the consumption of carbon anodes, and carbon anode slag is a common hazardous waste in the aluminum industry. In this work, electrolytic aluminum carbon anode slag was separated by flotation. Using the selectivity index (*SI*) as an indicator, the influencing factors of the carbon slag flotation process were optimized, and the separation performance of carbon and cryolite in the carbon anode slag was investigated. The raw carbon anode slag was ground for 40 min to achieve dissociation of the cryolite from the carbon, the optimized *SI* value was then used to determine the optimal flotation test conditions. The test results showed that the *SI* value under the optimal grinding flotation was approximately four times larger than the value of direct flotation. This indicated that carbon anode slag had a better flotation selectivity under the grinding flotation, which significantly improved the flotation performance.

## 1 Introduction

Cryolite-alumina molten salt electrolysis has been the most commonly used process for producing aluminum since its invention (Grjotheim, 1982). This process uses carbon as its electrolytic anode in industrial production. The carbon anode often bursts during electrolysis due to uneven combustion, and the carbon slag particles fall off from the anode surface and then come into the electrolyte thus affecting the production. At present, carbon anode slag from aluminum electrolysis is generally discarded as waste or used as fuel. Carbon anode slag is primarily composed of cryolite (Na_3_AlF_6_) and carbon, which are valuable raw materials for aluminum electrolysis, and carbon can also be used as raw materials for the preparation of porous carbon materials (Li et al., 2021). When the carbon anode slag is discarded directly, it will pollute the environment; when it is burned as fuel, it will waste the high extra value electrolyte. The comprehensive recovery and utilization of electrolytic aluminum spent carbon anode slag (Hereinafter simplified as “carbon anode slag”) are therefore necessary.

Flotation is a physical separation method used to separate particles by using the difference in surface property of particles (Chen et al., 2022; Ni et al., 2022). However, the flotation method is generally considered to be difficult to improve carbon purity and recovery efficiency, because cryolite are embedded during the process of carbon anode slag formation. In the mineral flotation, if there are different mineral embedding or locked bodies in the feed particles, grinding for mineral liberation is a prerequisite for a successful flotation separation (Li and Gao, 2017; Mathe et al., 2021). A grinding flotation process was proposed to separate and recover the LiCoO_2_ and graphite from spent lithium-ion batteries (Yu et al., 2018). The flotation performance of carbon anode slag through process optimization (a closed-circuit process of one roughing, one scavenging and two cleaning) was significantly improved (Mei et al., 2016). Li et al. (2021) studied the effects of particle size distribution of carbon anode slag, impeller speed, slurry concentration, reagent addition, and flotation time on the flotation performance of electrolysis aluminum carbon anode slag basing on the carbon recovery rate and separation flotation efficiency index. They concluded that the carbon recovery rate in the optimization experiment was increased by 1.7%, and the separation flotation efficiency index was improved by 4.1%. Grinding flotation performance is significantly affected by grinding parameters such as wet or dry conditions, grinding time, and grinding devices. Compared to dry-ground particles, wet-ground particles had more irregular shape factors and smoother surfaces, resulting in shorter induction times and higher floatation recovery rates (Bu et al., 2019). Ulusoy et al. researched the relationship between the shape, surface roughness values and wettability of various minerals (quartz, barite, and calcite) under different dry mills (ball, rod, and autogenous) (Ulusoy et al., 2003; Ulusoy and Yekeler, 2004, Ulusoy and Yekeler, 2007). Thus, for different minerals, shape and roughness affect surface hydrophobicity differently. Given this, there is no systematic study on grinding flotation of carbon anode slag.

Flotation kinetics results are often described using cumulative recovery *versus* time in batch flotation. Typically, the widely used and classical first-order kinetic model is applied to analyze the effects of different factors (i.e., emulsified reagent, salt ions, and operation parameters) on the flotation performance of the minerals (Bu et al., 2017a; Chen et al., 2019; Zheng et al., 2020; Zhou et al., 2020). In terms of the correlation of flotation kinetics for carbon anode slag, currently, fewer studies have examined the influence of different operating factors on flotation performance.

Therefore, in this study, the flotation performance of carbon anode slag under different factors (grinding time, flotation feed solid concentration, collector dosage, frother dosage, and pH of slurry) was investigated using selectivity index (*SI*), and optimal conditions for grinding flotation were optimized. As a next step, the flotation performance of direct flotation and optimal grinding flotation of carbon anode slag was compared based on the ash content of the flotation products. Finally, to compare the differences in the recovery rates of combustible materials (*R*_c_) and ash (*R*_a_) for concentrate, the kinetic analyses of direct flotation and optimal grinding flotation of carbon anode slag were carried out. Thus, the flotation selectivity of carbon anode slag under each method was evaluated.

## 2 Materials and methods

### 2.1 Materials

Carbon anode slag was obtained from Shandong Province, containing 87%–90% cryolite and 10%–13% carbon. After drying, the representative sub-samples, used in the whole experiments, were obtained through mixing the carbon anode slag by thorough coning and quartering. The carbon anode slag was sieved with the help of a RK/ZSP-*Φ*200 slapping sieve shaker (Wuhan Rock Crush & Grinding Equipment Manufacture Co., Ltd. Wuhan, China) to determine the particle size distribution. The carbon anode slag was characterized by X-Ray diffraction (XRD), X-Ray fluorescence (XRF), and field emission scanning electron microscopy (FESEM).

### 2.2 Methods

#### 2.2.1 Grinding experiments

The grinding tests were conducted under dry conditions using a QM-5 laboratory-scale ball mill (Changsha Tianchuang Powder Technology Co., Ltd. Changsha, China) equipped with a 2.0 L stainless steel cylinder (Diameter 12.5 cm and length 16.0 cm). A total of 2.22 kg of stainless steel balls were used as milling media. The diameters of the stainless steel balls were 1.20 cm, 0.90 cm, 0.62 cm, and 0.55 cm and their mass percentages were 33.75%, 31.40%, 18.37%, and 16.48%, respectively. The operational speed of the ball mill was set at 115 rpm (62.16% of critical rotational speed). The calculation of critical rotational speed for ball mill refers to the published literature (Ni et al., 2022). A mass of 200 g carbon anode slag with the size of −1 mm was fed into the ball mill.

#### 2.2.2 Flotation experiments

A 0.5 L RK/FD-II flotation machine (Wuhan Rock Crush & Grinding Equipment Manufacture Co., Ltd. Wuhan, China) was used for flotation tests with the impeller speed of 1900 rpm and 200 L/h of airflow rate. Kerosene and terpenic oil were used as collector and frother, respectively. The specific operation steps of flotation were as follows. First, carbon anode slag was pre-wetted and then poured into the flotation machine. Next, kerosene was added after 2 min of agitation, and then the slurry was mixed for 2 min. After which terpenic oil was added and the slurry was stirred for 0.5 min. Following this, the air was introduced, and after 0.5 min of aeration, scraping of froth was started. Flotation concentrates were collected at intervals of 0.5, 1, 2, 3, and 5 min. Following flotation, the concentrates and tailings were filtered, dried, weighed, and analyzed.

#### 2.2.3 XRD and XRF tests

The sample was ground to −0.074 mm using an HLXPM-*Φ*120 × 3 three-headed grinding machine (Wuhan Hengle Mineral Engineering Equipment Co., Ltd. Wuhan, China). The XRD and XRF tests were conducted with a D8 Advance X-Ray diffractometer (Bruker Company, Germany) and a ZSX Primus Ⅳ XRF (Rigaku Company, Japan), respectively. The tube current and voltage of XRD were 30 mA and 40 kV respectively. The anode target material used was Cu target, Kα radiation. The sample for XRD analysis was measured from 5 to 85° (2θ) with the scanning rate of 5°/min. The chemical composition of the carbon anode slag was acquired through XRF.

#### 2.2.4 FESEM measurement

A high-resolution FESEM (MA1A3 LMH, Tescan Company, Czech) equipped with an energy dispersive spectroscopy (EDS) was applied to analyze the surface morphology and element distribution of carbon anode slag. The FESEM was operated at an accelerating voltage of 10.0 kV and with secondary electron detector. Before the FESEM experiments, all samples were sputtered with the gold-palladium layer to promote the surface conductivity.

#### 2.2.5 Polarized light microscopy measurement

Thin sections were made from raw carbon anode slag, the product of carbon anode slag after 40 min, and optimal grinding flotation tailings. The particle size for the preparation of thin sections was + 45 μm. The embedding and liberation state between cryolite and carbon particles was then observed using the Polarizing microscope CX40P (Sunny optical instrument, Zhejiang, China).

### 2.3 Evaluation index of flotation

The first-order kinetic model, given in Eq. 1 (Sokolović and Miskovic, 2018), is used to obtain the parameters of *K* and *R*_∞_ through fitting the flotation test data. After that, the two parameters are used to evaluate the effect of the variables on flotation (Lynch et al., 1981). However, it is difficult to establish a trend for *K* and *R*_∞_ values under various conditions using this approach. Therefore, the modified rate constant (*K*_m_), defined as the product of *R*_∞_ and *K*, was given in Eq. 2 (Xu, 1998). Xu et al. defined the ratio of *K*_m_ between mineral I and mineral II as the selectivity index (*SI*) (Xu, 1998). The expression of *SI* is presented in Eq. 3. Since *K* and *R*_∞_ were comprehensively considered in the calculation of *SI*, some researchers have applied *SI* to compare the effects of different factors on flotation performance (Sripriya et al., 2003; Vapur et al., 2010; Marion et al., 2017; Zheng et al., 2020).

$$R=R_{\infty }.\left(1-e^{-K.t}\right)$$ (1)

Where, *R* is the combustible/ash matter recovery in %; *R*_∞_ is the ultimate percentage (%) recovery; *K* is the first-order rate constant (min^−1^), and *t* refers to the cumulative flotation time (min). MATLAB software was used to fit the first-order kinetic model to the flotation test results to obtain *R*_∞_ and *K* values. Coefficient of determination (*R* ^2^) was used to evaluate the accuracy of the model fit, and if it was higher than 0.8, Eq. 1 can be applied (Wang et al., 2021b).

$$K_{m}=R_{\infty }.K$$ (2)

$$SI\left(\frac{I}{II}\right)=\frac{K_{m}\;of\;mineral\;I}{K_{m}of\;mineral\;II}$$ (3)

Where, minerals Ι and ΙΙ represent the combustible and the ash materials in the flotation concentrate, respectively. The greater the *SI* value, the better the flotation selectivity. The expressions of *R*_c_ and *R*_a_ for flotation concentrate are shown in Eqs 4, 5, respectively.

$$R_{c}=\frac{\gamma_{c}.\left(100-A_{c}\right)}{100-A_{f}}$$ (4)

$$R_{a}=\frac{\gamma_{c}.A_{c}}{A_{f}}$$ (5)

Where, *γ*_c_ refers to the yield of flotation concentrate (%); *A*_c_ (%) and *A*_f_ (%) are the ash content of flotation concentrate and feed material, respectively.

## 3 Results and discussion

### 3.1 Characterization of raw carbon anode slag

**FIGURE 1:**
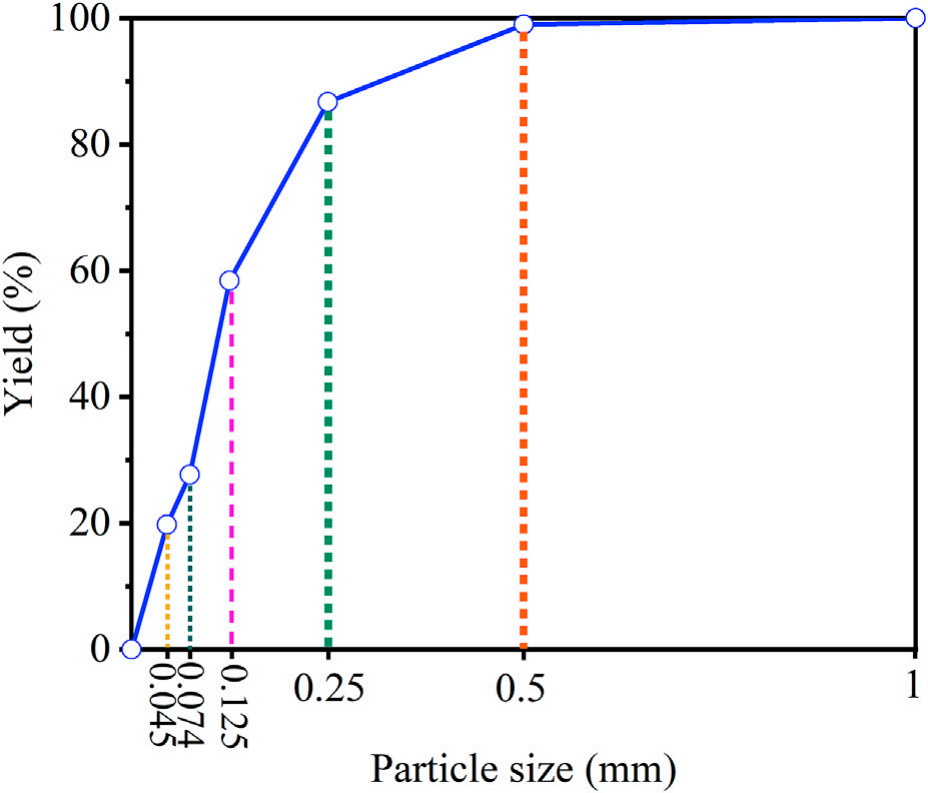
Cumulative particle size curve of carbon anode slag.

**FIGURE 2:**
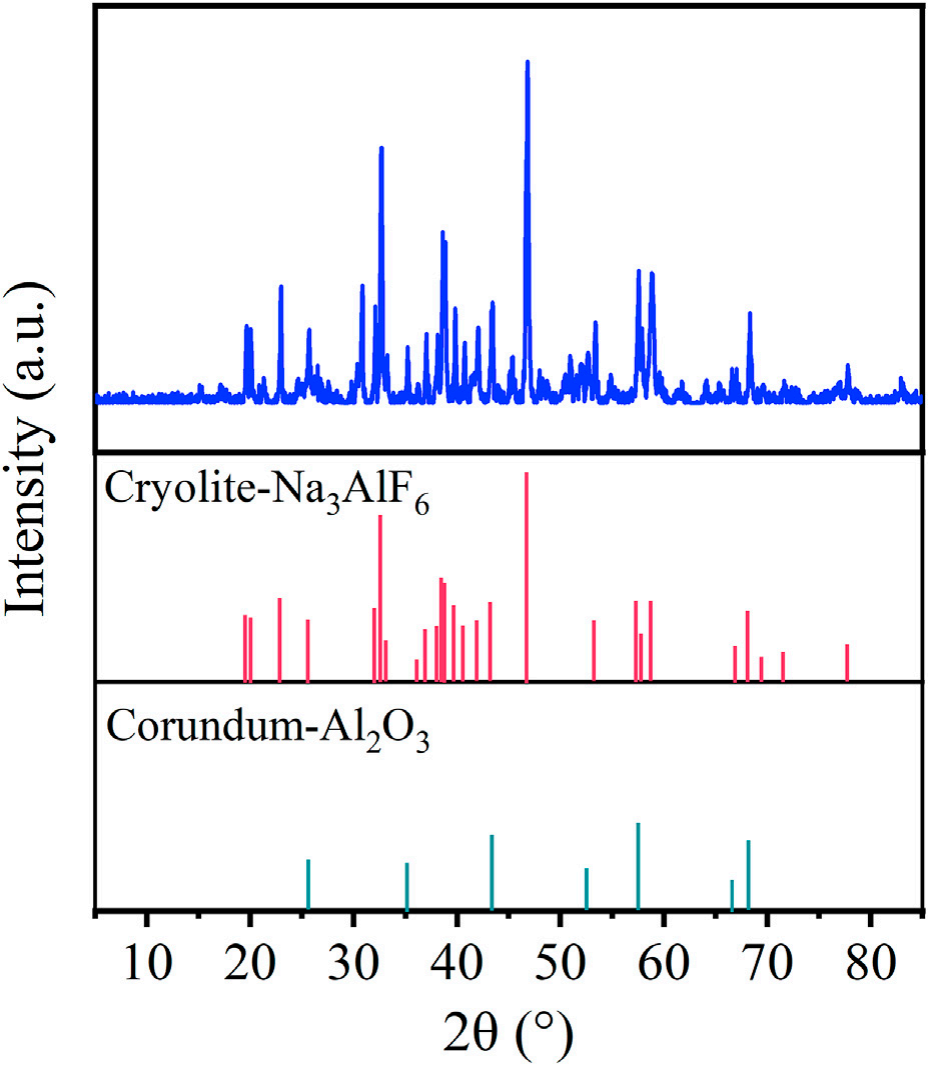
XRD pattern of carbon anode slag.

**FIGURE 3:**
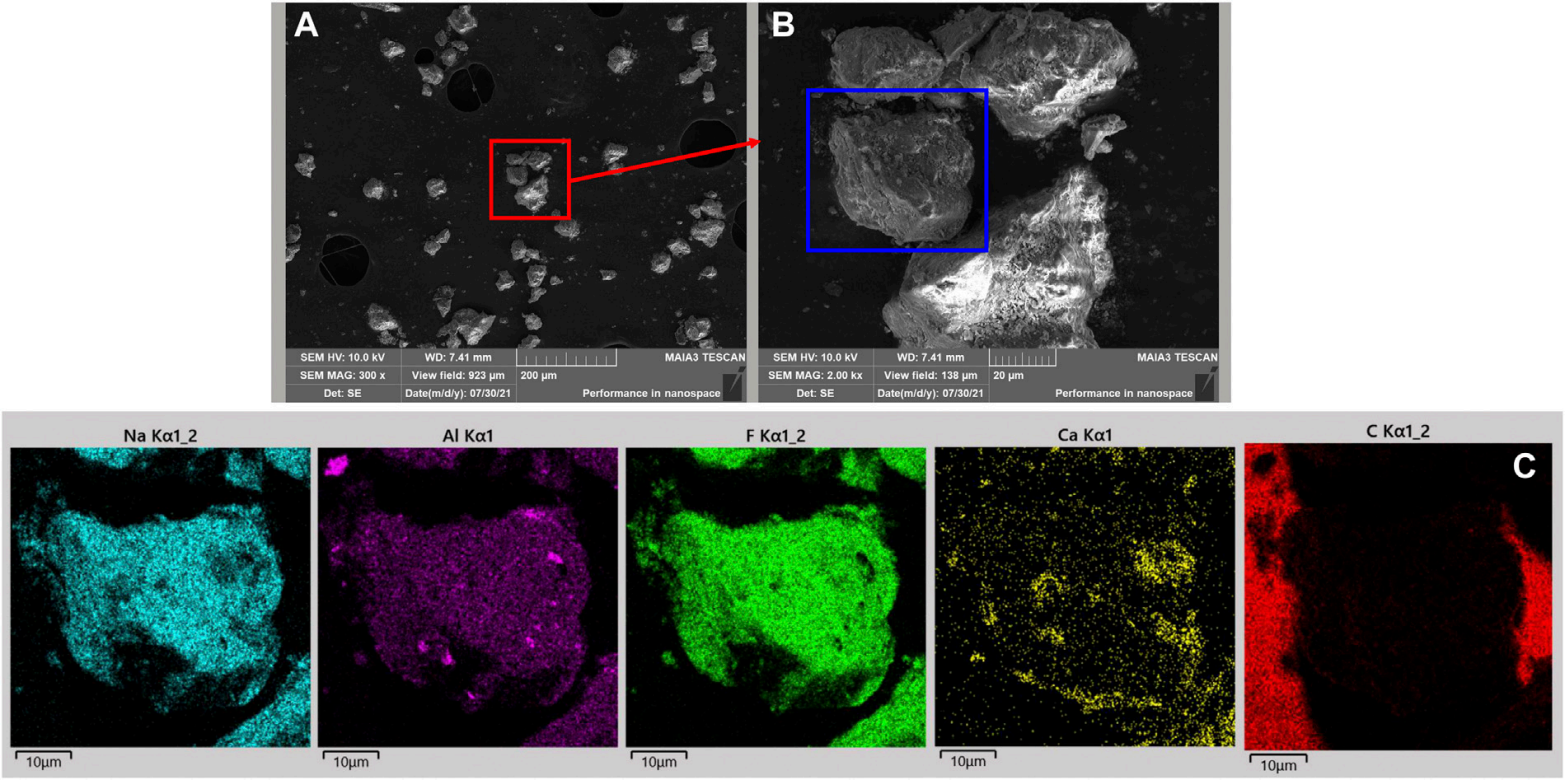
Images of **(A)**, **(B)** surface morphology and **(C)** element distribution in the carbon anode slag. Image **(B)** is an enlarged view of image **(A)** where is highlighted with a red rectangle. The area captured in image **(C)** was highlighted with a blue rectangle in image **(B)**.

The cumulative particle size result is shown in Figure 1. From Figure 1, the content of fraction size of 0.125 mm–0.074 mm (30.76%) was the most, followed by 0.25 mm–0.125 mm (28.29%). The content of −0.045 mm fraction size was 19.74%, and the proportion of this micro-fine fraction was small. The chemical composition is provided in Table 1. As given in Table 1, the major elements in the carbon anode slag were fluorine (F, 52.77%), sodium (Na, 22.10%), and aluminum (Al, 15.83%). The XRD pattern is illustrated in Figure 2. The mineral components of carbon anode slag contained cryolite and corundum, which were in agreement with the content of major elements determined by XRF. The surface morphology and element distribution images of carbon anode slag are demonstrated in Figure 3. As shown in Figures 3A,B, the particle shape was irregular, and the particle surface was rough. The distribution of Na, Al, and F elements in Figure 3C indicated that the component of carbon anode slag was mainly cryolite. Here, the C element detected by EDS was primarily the C element on the conductive tape.

**TABLE 1:** Element components from XRF analysis.

Element	F	Na	Mg	Al	Si	S	K	Ca
wt (%)	52.77	22.10	0.27	15.83	0.10	0.39	0.19	2.54

### 3.2 Effect of different factors on flotation performance of carbon anode slag

#### 3.2.1 Grinding time

**FIGURE 4:**
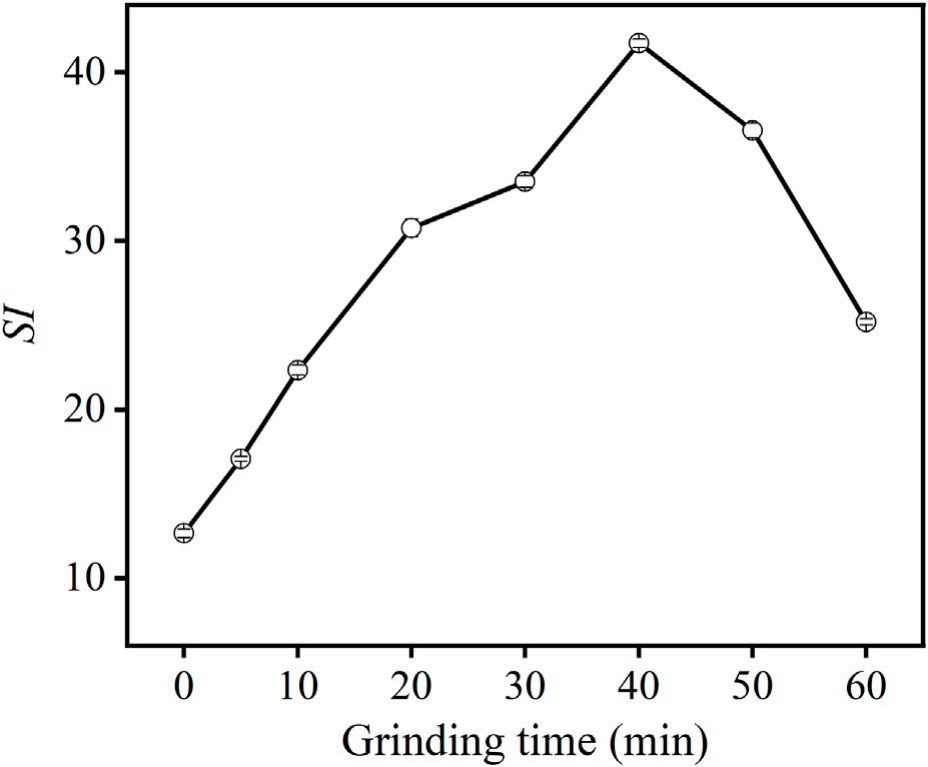
Effect of grinding time on *SI* of carbon anode slag flotation (feed solid concentration 200 g/L, colletor dosage 1,000 g/t, frother dosage 500 g/t).

To improve flotation performance, the cryolite bound to carbon in carbon anode slag must be ground to be liberated from carbon. Therefore, carbon anode slag was ground before flotation to investigate the effect of grinding time on flotation performance. Figure 4 illustrates the effect of grinding time on the *SI* of carbon anode slag. From Figure 4, when the grinding time increased, the *SI* value first increased, then decreased. When the grinding time was 40 min, the *SI* value reached the maximum value of 41.89, which was about three times that of 0 min grinding time (i.e., direct flotation of carbon anode slag). These results indicate that grinding pretreatments significantly improved flotation recovery; because the carbon and cryolite separated in the carbon anode slag after grinding. When the grinding time was shorter than 40 min, the particles were dissociated to a certain extent, but they were also prone to detachment in the flotation process due to their large gravity, which did not permit the recovery of carbon particles. When the grinding time was longer than 40 min, carbon anode slag was over-grinding, thus causing more micro-cryolite particles to enter the froth concentrate (Wang et al., 2021a). Floatability was poor due to the excessive fine particles. The optimal grinding time was determined to be 40 min. Therefore, the subsequent flotation experiments were performed on the carbon anode slag after 40 min grinding treatment.

**FIGURE 5:**
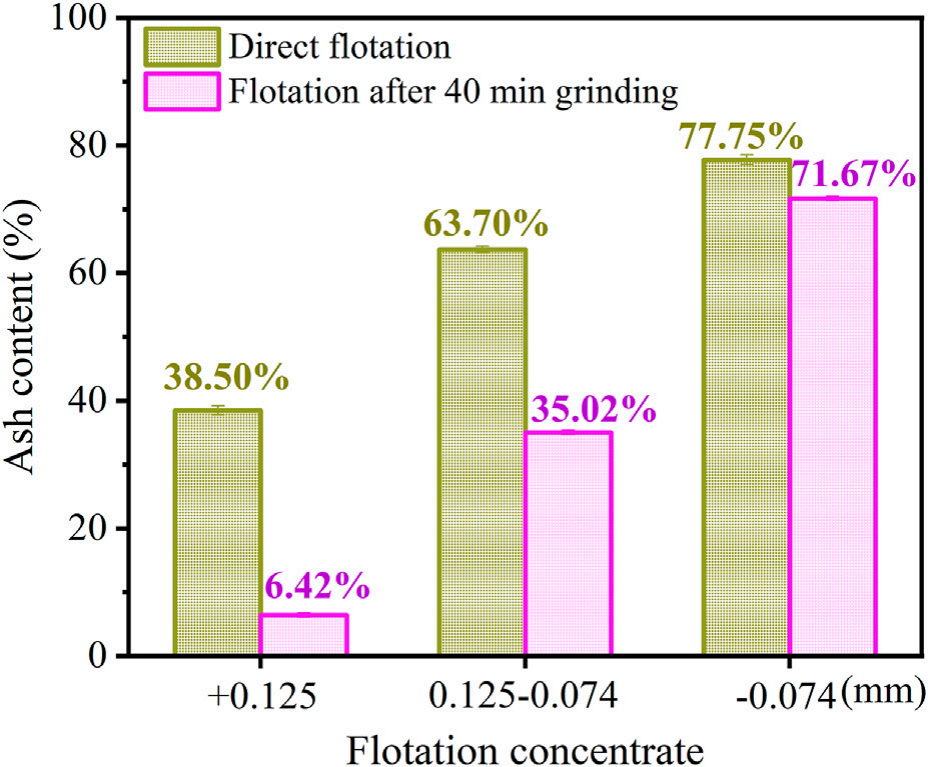
Comparison diagram of ash content of flotation concentrate with different sizes under different methods.

**FIGURE 6:**
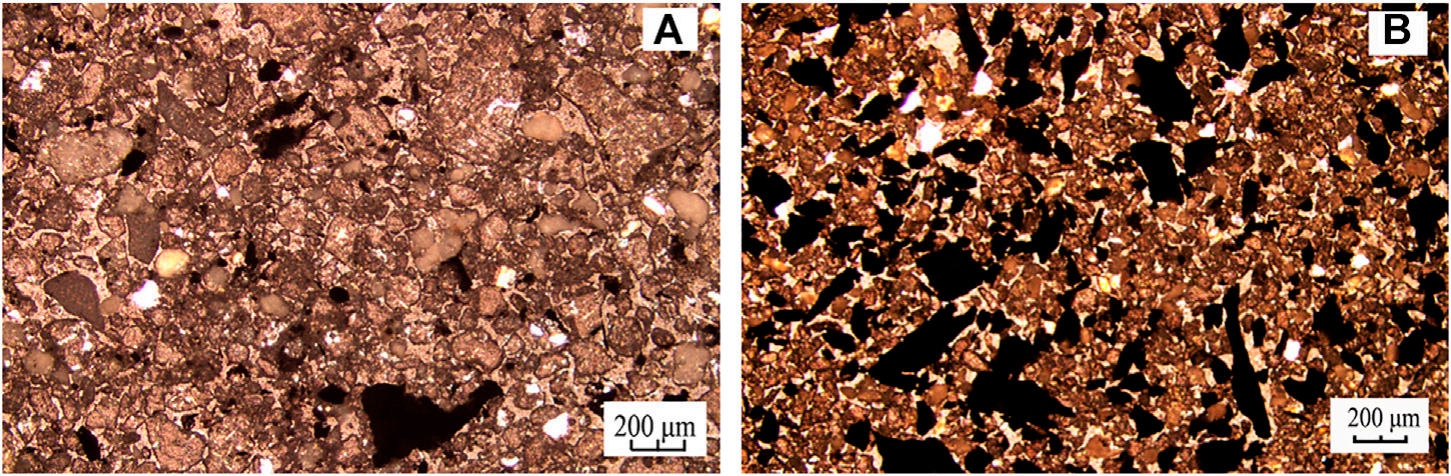
Microscope photographs of thin sections of **(A)** raw carbon anode slag and **(B)** the product of carbon anode slag after 40 min grinding.

The ash content of flotation concentrate with different sizes under direct flotation and flotation after 40 min grinding treatment of carbon anode slag is illustrated in Figure 5. From Figure 5, it can be seen that the ash content of + 0.125 mm and 0.125–0.074 mm in the concentrate under the flotation after 40 min grinding treatment were 6.42% and 35.02%, respectively, which were significantly lower than that of the corresponding particle size in the direct flotation. It indicated that the grinding treatment had effectively separated the cryolite adhering to the coarser carbon particles, thus improving the flotation selectivity of the coarser carbon particles and promoting the flotation process. The microscope observations of the raw carbon anode slag and the product of carbon anode slag after 40 min grinding were demonstrated in Figure 6. Comparing Figures 6A,B, it is found that there were significantly more black carbon particles in the product after grinding treatment than in the raw carbon anode slag, indicating that cryolite particels were enriched in the fine-grainded fraction; carbon particles were enriched in the coarse-grainded fraction. This was because there was a difference in hardness between cryolite and carbon particles, which led to the selective crushing of carbon anode slag in the grinding process (Ni et al., 2022).

#### 3.2.2 Effects of feed solid concentration

**FIGURE 7:**
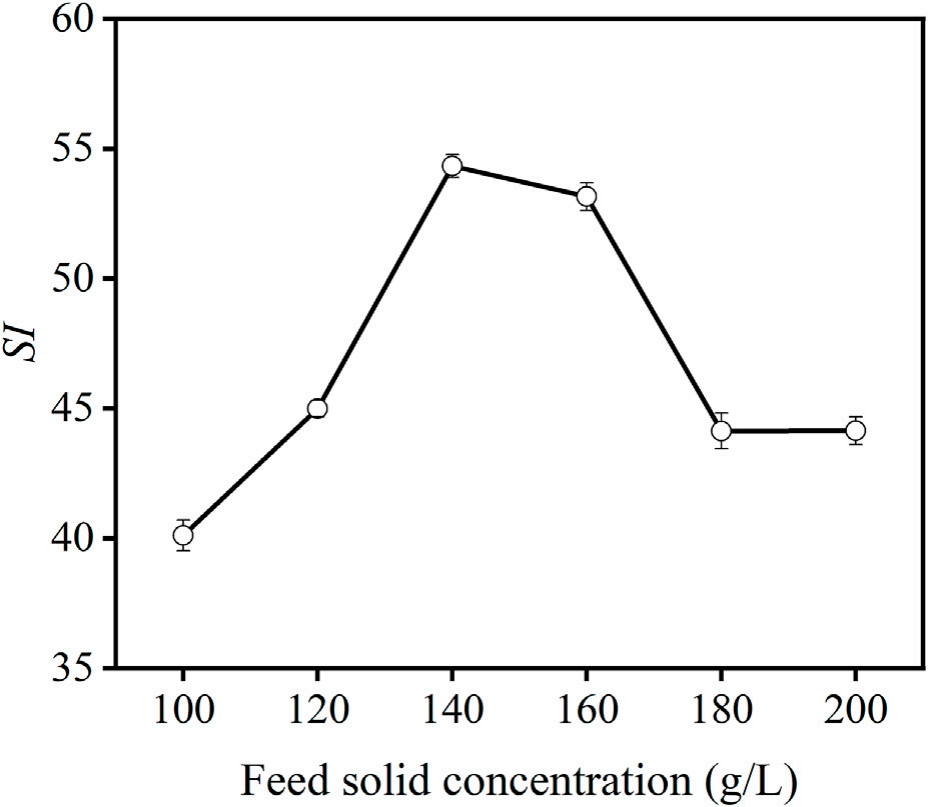
Effect of feed solid concentration on *SI* of carbon anode slag flotation (grinding time 40 min, colletor dosage 1,000 g/t, frother dosage 500 g/t).

The effects of feed solid concentration on the flotation performance are mainly reflected in the change of flotation time, the “excess” and “insufficient” flotation reagent, and the change of aeration rate, which directly affects the recovery and ash content of the concentrate. Figure 7 shows the effect of feed solid concentration on the flotation performance of carbon anode slag. As can be seen in Figure 7, the *SI* value tended to increase and then decrease as the feed solid concentration increased, and the *SI* reached a maximum value at a feed solid concentration of 140 g/L.

When the feed solid concentration was lower (<140 g/L), the *SI* value increased with the increase in the feed solid concentration, which is consistent with the literature (Luo et al., 2016; Bu et al., 2017b). The increase in the solid concentration is helpful for the collison between bubbles and particles, which leads to the improvement in the flotation recovery of valuable particles. When the feed solid concentration was higher than 140 g/L, the reduction of *SI* was observed. This could be attributed to the detachment of bubbles from particle surfaces and also reduction of bubble numbers with increasing of pulp density for a given air flow rate (Mowla et al., 2008). Furthermore, the flotation time of the particles was relatively long, and there existed entrainment of more micro-cryolite particles, in turn leading to a lower *SI* (Wang et al., 2015). Therefore, 140 g/L was selected as the optimal value for feed solid concentration.

#### 3.2.3 Collector dosage

**FIGURE 8:**
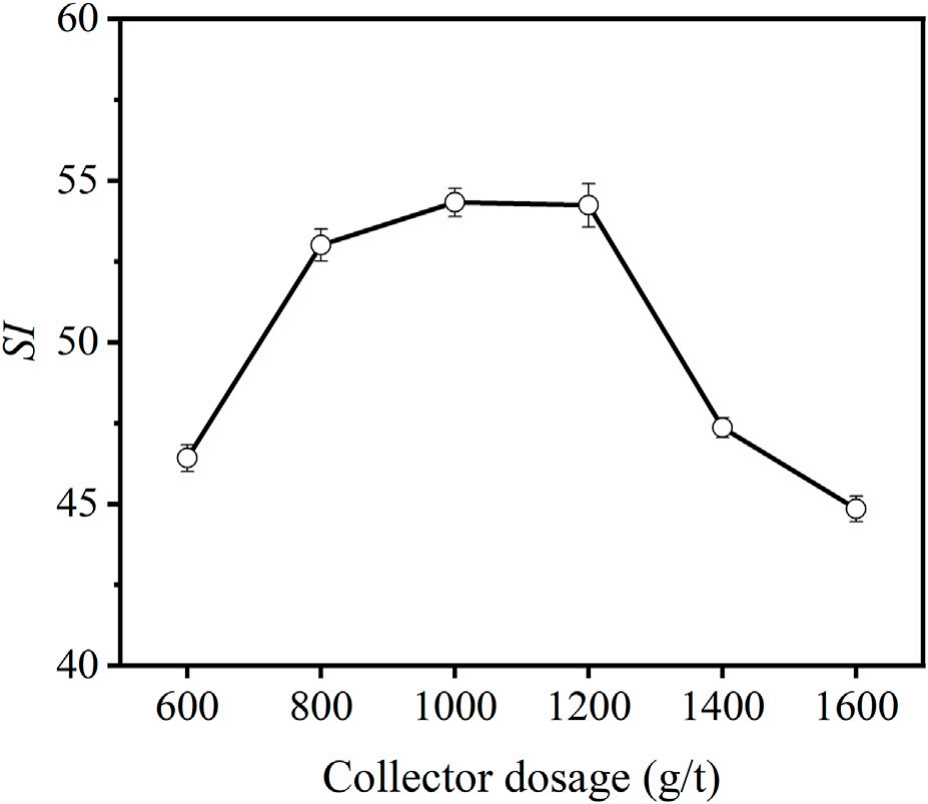
Effect of collector dosage on *SI* of carbon anode slag flotation (grinding time 40 min, feed solid concentration 140 g/L, frother dosage 500 g/t).

The effect of collector dosage on *SI* is illustrated in Figure 8. From Figure 8, when the collector dosage increased from 600 g/t to 1,000 g/t, the *SI* showed an increasing trend; when the dosage of the collector continued to increase, the *SI* value showed a decreasing trend. When the dosage of the collector was not enough, the collector’s ability to capture particles was inadequate. When the collector dosage was gradually increased, the hydrophobic particles were promoted into the froth concentrate, thus increasing *SI*. When the collector dosage was 1,000 g/t, the *SI* value reached its maximum. When the collector dosage was >1,000 g/t, due to the excessive collector dosage, solid particle selectivity in the slurry was poor, resulting in a decrease in *SI* (Bu et al., 2018). Further more, oil droplet can promote the liquid drainage of froth, leading to bubble coalescence and breakup (Zhou et al., 2020). Thus, the poor flotation performance at excessively high collector dosage can be due to the poor froth stability resulting from the oil droplets.

#### 3.2.4 Effects of frother dosage

**FIGURE 9:**
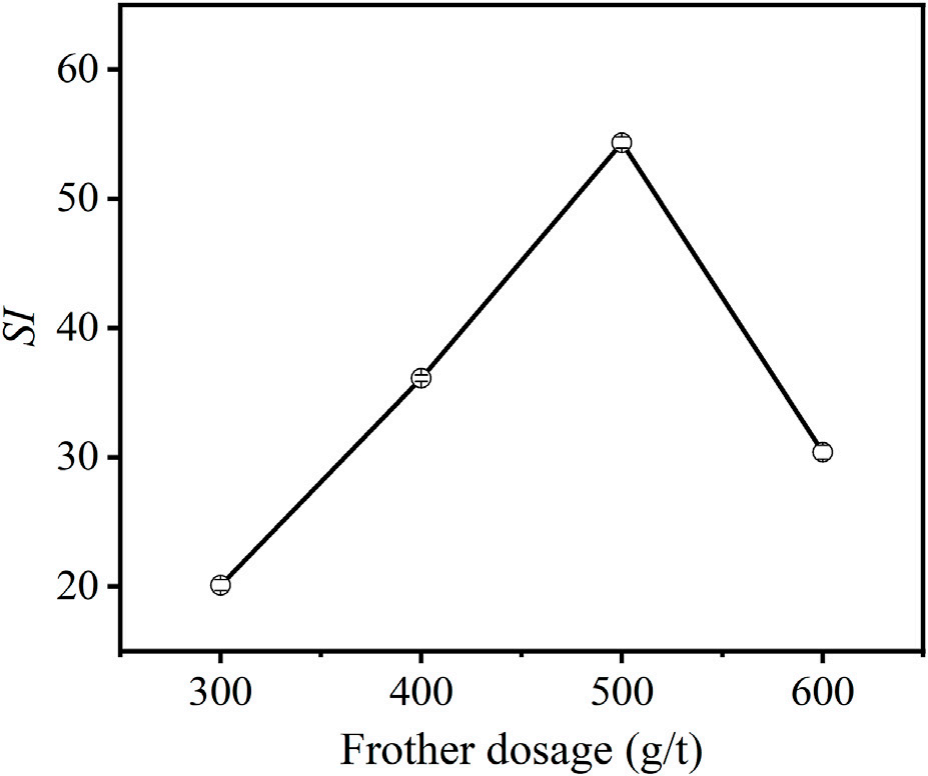
Effect of frother dosage on *SI* of carbon anode slag flotation (grinding time 40 min, feed solid concentration 140 g/L, collector dosage 1,000 g/t).

Figure 9 provides the effect of frother dosage on the flotation performance of carbon anode slag. As can be seen from Figure 9, *SI* values increased with increasing frother dosage, followed by decreasing trends. The low *SI* value at low frother dosage is due to the poor bubble stability. With the increasing frother dosage, the froth become stable, which is helpful for the collection of mineralized bubble *via* the froth zone (Farrokhpay, 2011). At a dosage of 500 g/t, the *SI* reached its maximum value. Increasing the frother dosage over 500 g/t resulted in more froth entrainment due to the sticky froth of terpenic oil, so the *SI* value decreased again (Nguyen and Schultze, 2004). Therefore, 500 g/t was chosen as the optimal froth dosage.

#### 3.2.5 pH of the slurry

**FIGURE 10:**
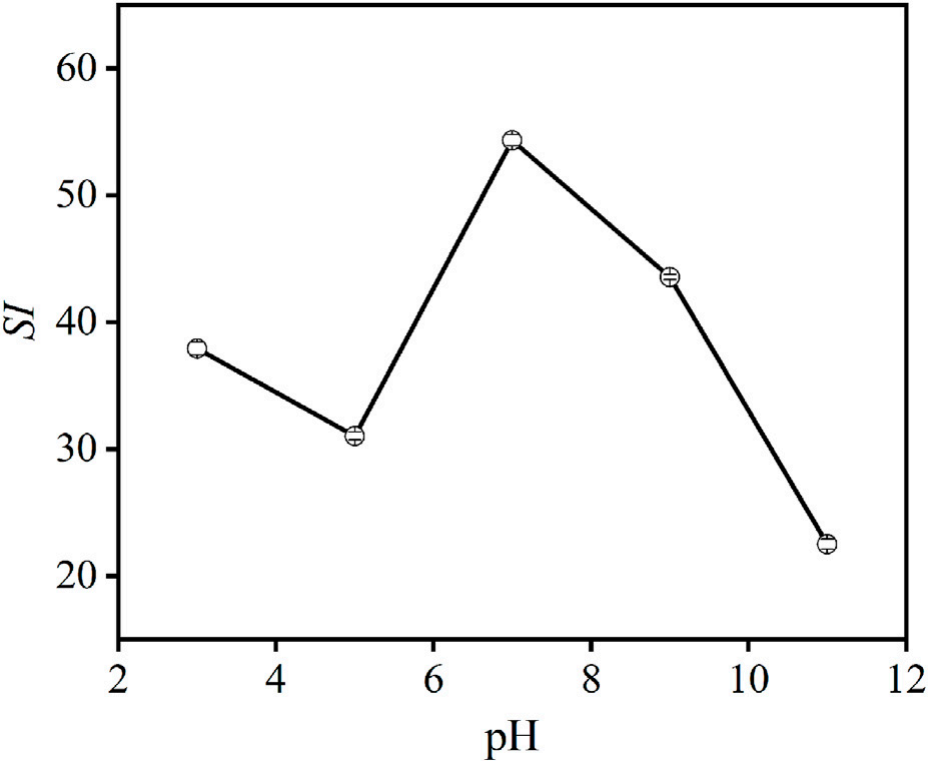
Effect of pH on *SI* of carbon anode slag flotation (grinding time 40 min, feed solid concentration 140 g/L, collector dosage 1,000 g/t, frother dosage 500 g/t).

The effect of slurry pH on the flotation performance of carbon anode slag is illustrated in Figure 10. It can be seen that, when pH is <7 or >7, the *SI* value is decreased in both cases, indicating that when the flotation slurry was acidic or alkaline, it was not conducive to the flotation of carbon anode slag, thus resulting in poor flotation performance. This is because the molecular activity of the surfactant was inhibited by acidic or alkaline solution environments, thereby reducing the flotation selectivity of carbon anode slag. Accordingly, pH seven was determined to be the optimal pH of the flotation slurry.

The optimal experiment conditions for grinding flotation of carbon anode slag were: grinding time 40 min, feed solid concentration 140 g/L, pH 7, collector dosage 1,000 g/t, and frother dosage 500 g/t.

### 3.3 Comparative analysis of flotation performance

#### 3.3.1 Ash content comparison of flotation products under different periods

**FIGURE 11:**
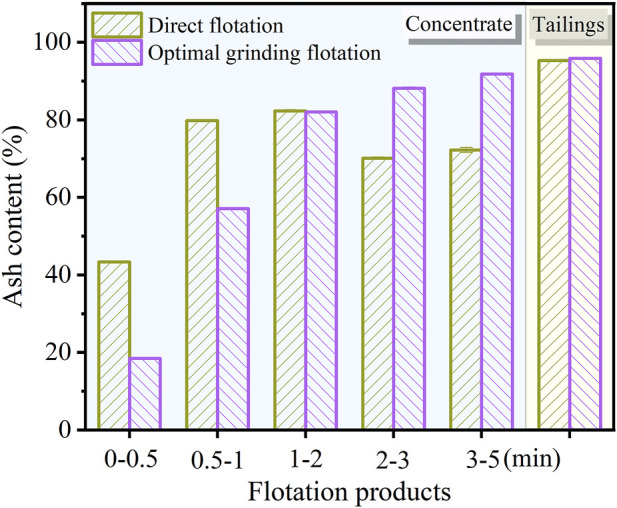
Comparison diagram of ash content of flotation products under different periods.

The ash content of carbon anode slag flotation products under direct flotation and optimal grinding flotation is shown in Figure 11. According to Figure 11, the ash content of carbon collected in 0–0.5 min and 0.5–1 min under the optimal grinding flotation was significantly lower than that direct flotation, because the carbon and cryolite in carbon anode slag were effectively dissociated after grinding treatment. Tailings obtained by this method had a higher ash content than tailings obtained by direct flotation. In the flotation process, carbon particles are recovered in the early stages and cryolite (hydrophobic particles) are recovered in the later stage due to entrainment. Thus, under the direct flotation, the carbon anode slag was not dissociated, and the locked bodies of cryolite and carbon were collected at the stage of 2–3 min and 3–5 min, resulting in lower ash content of concentrate than that under the optimal grinding flotation. This revealed that when carbon anode slag was ground and floated, the dissociated carbon particles mainly floated out during the periods 0–0.5 min and 0.5–1 min, resulting in good separation efficiency.

#### 3.3.2 Analysis of microscope photographs

**FIGURE 12:**
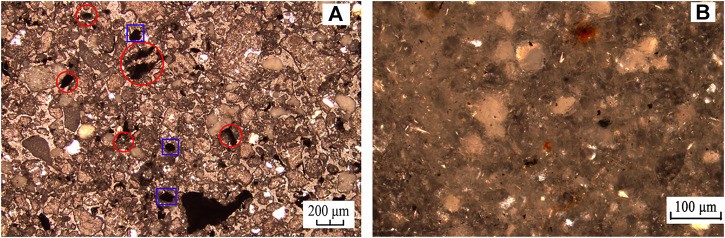
Microscope photographs of thin sections of **(A)** raw carbon anode slag and **(B)** tailings of optimal grinding flotation.

Figure 12 shows the microscope observations of the raw carbon anode slag and the tailings under the optimal grinding flotation. In Figure 12A, the black particles marked by blue box lines were monomeric carbon particles, and the particles marked by red box lines were carbon and cryolite in an embedded state. It indicated that there were more locked bodies of carbon and cryolite and less monomeric carbon, further dissociation of locked bodies of carbon and cryolite can improve carbon anode slag flotation performance. As can be seen from Figure 12B, there were fewer black carbon particles in the flotation tailings, demonstrating that the effective separation of carbon and cryolite particles can be achieved through grinding treatment. After grinding, the carbon and cryolite were sufficiently dissociated, and the carbon was enriched into the froth concentrate, thereby improving the cryolite tailings ash content. As a result, it was confirmed that optimal grinding flotation of carbon anode slag produced a better flotation performance than direct flotation.

### 3.4 Comparison of the recovery rate of flotation concentrate

**FIGURE 13:**
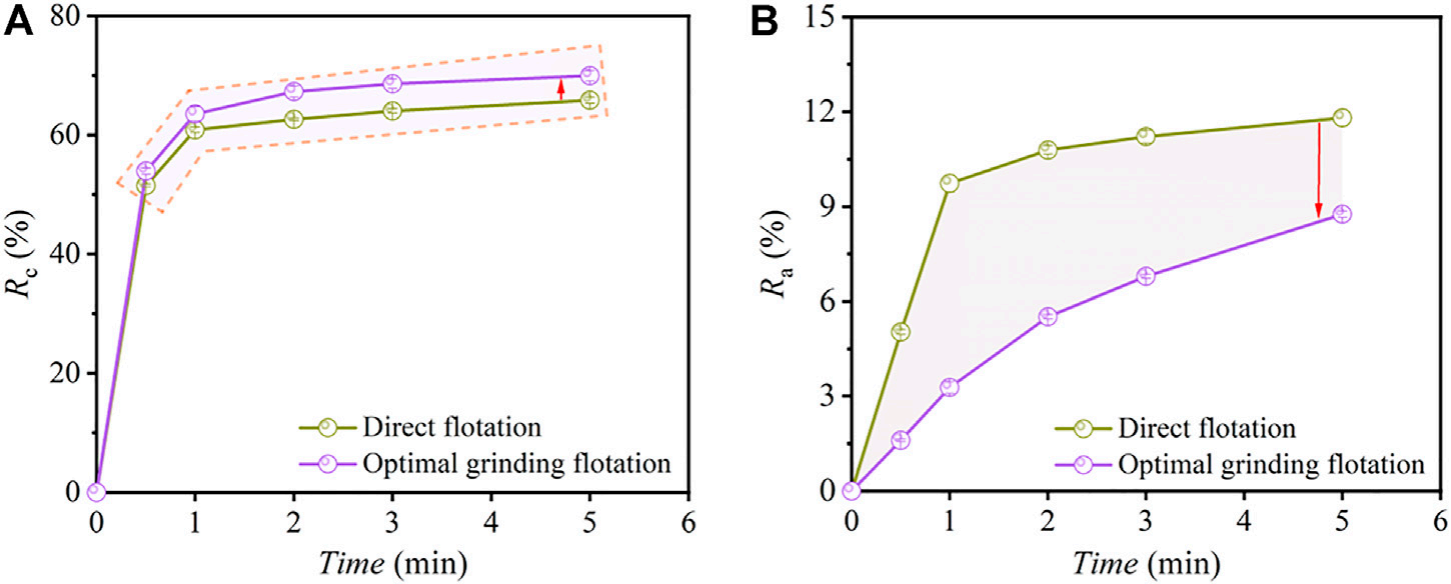
*R*_c_ **(A)** and *R*_a_ **(B)** of concentrate *versus* time under the methods of direct flotation and optimal grinding flotation.

The recovery rate of combustible materials (*R*_c_) and ash materials (*R*_a_) of concentrate (direct flotation vs. optimal grinding flotation) as a function of time is shown in Figure 13. According to Figure 13A, the *R*_c_ of the concentrate at 0.5–5 min under the method of optimal grinding flotation was higher than that of direct flotation, indicating that the carbon could be recovered from carbon anode slag efficiently through grinding treatment before flotation. As shown in Figure 13B, the *R*_a_ of the concentrate for optimal grinding flotation was lower than that of direct flotation. Moreover, under the grinding flotation method, the ash content in the concentrate was significantly reduced, and micro-cryolite particles were significantly reduced from entering the froth concentrate. Table 2 gives the *R*_∞_ and *K* values of flotation concentrate combustible/ash materials for each method. As can be seen in Table 2, the *SI* values for direct flotation and optimal grinding flotation were 12.82 and 54.64, respectively, and the latter was approximately four times larger than the former; indicating that grinding flotation has a better flotation selectivity for carbon anode slag than direct flotation.

**TABLE 2:** Output of non-linear regression fitting to first-order rate equation for a recovery rate of combustible and ash materials for flotation concentrate.

Flotation method	Combustible material				Ash material				*SI*
	*K* (min^−1^)	*R*_c∞_ (%)	*R* ^2^	*K*_m_	*K* (min^−1^)	*R*_a∞_ (%)	*R* ^2^	*K*_m_	
Direct flotation	3.22	63.77	0.9985	205.21	1.39	11.53	0.9855	16.00	12.82
Optimal grinding flotation	3.03	67.68	0.9980	204.73	0.36	10.42	0.9993	3.75	54.64

## 4 Conclusion

The effects of grinding time, flotation feed solid concentration, collector dosage, frother dosage, and pH of slurry on the flotation performance of carbon anode slag were investigated, and the flotation selectivity of carbon anode slag under the methods of direct flotation and grinding flotation was analyzed and compared. The following conclusions were drawn.

1) Carbon anode slag flotation separation is affected by all five factors. Based on the optimization value of *SI*, the optimal test conditions for grinding flotation were determined as follows: grinding time 40 min, feed solid concentration 140 g/L, collector dosage 1,000 g/t, frother dosage 500 g/t, and pH of slurry 7.
2) Under the optimal grinding flotation, the separated carbon particles were mainly recovered at the stages of 0–0.5 min and 0.5–1 min. This led to a better flotation performance for the carbon anode slag because the carbon and cryolite were separated.
3) Under grinding flotation, carbon anode slag had a higher recovery rate of combustible material in the concentrate than direct flotation.
4) The *SI* value of carbon anode slag under the optimal grinding flotation was approximately four times larger than the direct flotation. Consequently, carbon anode slag showed better flotation selectivity under the grinding flotation than direct flotation.

## Data Availability

The original contributions presented in the study are included in the article/Supplementary Material, further inquiries can be directed to the corresponding author.

## References

[B1] Bu X. Xie G. Peng Y. Ge L. Ni C. (2017a). Kinetics of flotation. Order of process, rate constant distribution and ultimate recovery. Physicochem. Probl. Mineral. Pro. 53 (1), 342–365. 10.5277/ppmp170128

[B2] Bu X. Zhang T. Chen Y. Xie G. Peng Y. (2017b). Comparative study of conventional cell and cyclonic microbubble flotation column for upgrading a difficult-to-float Chinese coking coal using statistical evaluation. Int. J. Coal Prep. Util. 40 (6), 359–375. 10.1080/19392699.2017.1359577

[B3] Bu X. Zhang T. Chen Y. Peng Y. Xie G. Wu E. (2018). Comparison of mechanical flotation cell and cyclonic microbubble flotation column in terms of separation performance for fine graphite. Physicochem Probl. Miner. 54 (3), 732–740.

[B4] Bu X. Chen Y. Ma G. Sun Y. Ni C. Xie G. (2019). Differences in dry and wet grinding with a high solid concentration of coking coal using a laboratory conical ball mill: Breakage rate, morphological characterization, and induction time. Adv. Powder Technol. 30, 2703–2711. 10.1016/j.apt.2019.08.016

[B5] Chen Y. Bu X. Truong V. N. T. Peng Y. Xie G. (2019). Study on the effects of pre-conditioning time on the floatability of molybdenite from the perspective of cavitation threshold. Min. Eng. 141, 105845. 10.1016/j.mineng.2019.105845

[B6] Chen Y. Li P. Bu X. Chehreh Chelgani S. Kong Y. Liang X. (2022). Resource utilization strategies for spent pot lining: A review of the current state. Sep. Purif. Technol. 300, 121816. 10.1016/j.seppur.2022.121816

[B7] Farrokhpay S. (2011). The significance of froth stability in mineral flotation--a review. Adv. Colloid Interface Sci. 166 (1-2), 1–7. 10.1016/j.cis.2011.03.001 21470589

[B8] Grjotheim K. (1982). Aluminium electrolysis: fundamentals of the Hall-Héroult process. Incorporated. Düsseldorf: Aluminium-Verlag.

[B9] Li C. Gao Z. (2017). Effect of grinding media on the surface property and flotation behavior of scheelite particles. Powder Technol. 322, 386–392. 10.1016/j.powtec.2017.08.066

[B10] Li B. Zhou J. Yao Z. Peng Q. Liu M. Li X. (2021a). Advances in the safe disposal and comprehensive utilization of spent carbon anode from aluminum electrolysis: Prospects for extraction and application of carbon resources from hazardous waste. Front. Energy Res. 9, 1–10. 10.3389/fenrg.2021.779476

[B11] Li H. Wang J. Hou W. Li M. Cheng B. Feng Y. (2021b). The study of carbon recovery from electrolysis aluminum carbon dust by froth flotation. Metals 11 (1), 145. 10.3390/met11010145

[B12] Luo X. Feng B. Wong C. Miao J. Ma B. Zhou H. (2016). The critical importance of pulp concentration on the flotation of galena from a low grade lead–zinc ore. J. Mat. Res. Technol. 5 (2), 131–135. 10.1016/j.jmrt.2015.10.002

[B13] Lynch A. J. Johnson N. Manlapig E. Thorne C. (1981). Mineral and coal flotation circuits: Their simulation and control. Amsterdam: Elsevier.

[B14] Marion C. Jordens A. Li R. Rudolph M. Waters K. E. (2017). An evaluation of hydroxamate collectors for malachite flotation. Sep. Purif. Technol. 183, 258–269. 10.1016/j.seppur.2017.02.056

[B15] Mathe E. Cruz C. Lucay F. A. Gálvez E. D. Cisternas L. A. (2021). Development of a grinding model based on flotation performance. Min. Eng. 166, 106890. 10.1016/j.mineng.2021.106890

[B16] Mei X. Li J. Yu Z. (2016). The research on recycling carbon residue by floatation process. Light Met. 4, 28–30.

[B17] Mowla D. Karimi G. Ostadnezhad K. (2008). Removal of hematite from silica sand ore by reverse flotation technique. Sep. Purif. Technol. 58 (3), 419–423. 10.1016/j.seppur.2007.08.023

[B18] Nguyen A. V. Schultze H. J. (2004). Colloidal science of flotation. New York: Marcel Dekker.

[B19] Ni C. Zhou S. Gao J. Bu X. Chen Y. Alheshibri M. (2022). Selective comminution and grinding mechanisms of spent carbon anode from aluminum electrolysis using ball and rod mills. Physicochem Probl. Miner. 58 (3), 1–15.

[B20] Sokolović J. Miskovic S. (2018). The effect of particle size on coal flotation kinetics: A review. Physicochem. Probl. Miner. Process. 54. 10.5277/PPMP18155

[B21] Sripriya R. Rao P. Choudhury B. R. (2003). Optimisation of operating variables of fine coal flotation using a combination of modified flotation parameters and statistical techniques. Int. J. Min. Process. 68 (1-4), 109–127. 10.1016/s0301-7516(02)00063-7

[B22] Ulusoy U. Yekeler M. (2004). Variation of critical surface tension for wetting of minerals with roughness determined by Surtronic 3+ instrument. Int. J. Min. Process. 74 (1), 61–69. 10.1016/j.minpro.2003.09.001

[B23] Ulusoy U. Yekeler M. (2007). Flotability of barite particles with different shape and roughness. Indian J. Chem. Technol. 14 (6), 616–625.

[B24] Ulusoy U. Yekeler M. Hicyilmaz C. (2003). Determination of the shape, morphological and wettability properties of quartz and their correlations. Min. Eng. 16 (10), 951–964. 10.1016/j.mineng.2003.07.002

[B25] Vapur H. Bayat O. Uçurum M. (2010). Coal flotation optimization using modified flotation parameters and combustible recovery in a Jameson cell. Energy Convers. Manag. 51 (10), 1891–1897. 10.1016/j.enconman.2010.02.019

[B26] Wang L. Peng Y. Runge K. Bradshaw D. (2015). A review of entrainment: Mechanisms, contributing factors and modelling in flotation. Min. Eng. 70, 77–91. 10.1016/j.mineng.2014.09.003

[B27] Wang X. Bu X. Alheshibri M. Bilal M. Zhou S. Ni C. (2021a). Effect of scrubbing medium’s particle size distribution and scrubbing time on scrubbing flotation performance and entrainment of microcrystalline graphite. Int. J. Coal Prep. Util. 163, 1–22. 10.1080/19392699.2021.1932843

[B28] Wang X. Shaoqi Z. Bu X. Ni C. Xie G. Peng Y. (2021b). Investigation on interaction behavior between coarse and fine particles in the coal flotation using focused beam reflectance measurement (FBRM) and particle video microscope (PVM). Sep. Sci. Technol. 56 (8), 1418–1430. 10.1080/01496395.2020.1777428

[B29] Xu M. (1998). Modified flotation rate constant and selectivity index. Min. Eng. 11 (3), 271–278. 10.1016/s0892-6875(98)00005-3

[B30] Yu J. He Y. Ge Z. Li H. Xie W. Wang S. (2018). A promising physical method for recovery of LiCoO 2 and graphite from spent lithium-ion batteries: Grinding flotation. Sep. Purif. Technol. 190, 45–52. 10.1016/j.seppur.2017.08.049

[B31] Zheng K. Bu X. Zhou S. Zhang J. Shao H. Sha J. (2020). Effects of monovalent and divalent ions in coal gasification brine on the froth entrainment and flotation kinetics of anthracite coal. Physicochem. Probl. Min. Process. 56 (5), 960–974. 10.37190/ppmp/127501

[B32] Zhou S. Wang X. Bu X. Shao H. Hu Y. Alheshibri M. (2020). Effects of emulsified kerosene nanodroplets on the entrainment of gangue materials and selectivity index in aphanitic graphite flotation. Min. Eng. 158, 106592. 10.1016/j.mineng.2020.106592

